# Investigating the Intercellular Communication Network of Immune Cell in Acute Respiratory Distress Syndrome with Sepsis

**DOI:** 10.1155/2022/4586648

**Published:** 2022-02-16

**Authors:** Pei Tao, Jinzhou He, Tao Ai, Yinghong Fan, Wei Zeng

**Affiliations:** ^1^Chengdu Women's and Children's Central Hospital, School of Medicine, University of Electronic Science and Technology of China, Sichuan 611731, China; ^2^Department of Pediatrics, Guangyuan Central Hospital, Sichuan 628000, China; ^3^Department of Hepatobiliary Surgery, Daping Hospital, Army Medical University, Chongqing 400042, China

## Abstract

Acute respiratory distress syndrome (ARDS) is recognized as a serious public health issue that results in respiratory failure and high mortality rates. The syndrome is characterized by immune cell aggregation, communication, activation, and alveolar epithelial damage. To elucidate the complex dynamic process of the immune system's response in ARDS, we construct the intercellular communication network of immune cells in ARDS based on a single-cell RNA sequencing dataset (including three sepsis-induced ARDS patients and four sepsis-only patients). The results show that macrophages relayed most of the intercellular signals (ligand–receptor pairs) in both groups. Many genes related to immune response (IFI44L, ISG, and HLA-DQB1) and biological functions (response to virus, negative regulation of viral life cycle, and response to interferon-beta) were detected via differentially expressed gene analysis of macrophages between the two groups. Deep analysis of the intercellular signals related to the macrophage found that sepsis-induced ARDS harbored distinctive intercellular signals related to chemokine–chemokine receptors (CCL3/4/5−CCR1), which mainly are involved in the disturbance of the STAT family transcription factors (TFs), such as STAT2 and STAT3. These signals and downstream TFs might play key roles in macrophage M1/M2 polarization in the process of sepsis-induced ARDS. This study provides a comprehensive view of the intercellular communication landscape between sepsis and sepsis-induced ARDS and identifies key intercellular communications and TFs involved in sepsis-induced ARDS. We believe that our study provides valuable clues for understanding the immune response mechanisms of ARDS.

## 1. Introduction

Acute respiratory distress syndrome (ARDS) is the most severe form of acute lung injury, comprising up to 10% of intensive care unit admissions [1]. With slow improvement, the high mortality rate decreased from 60% to 40% in the last 20 years [2, 3]. Further, survivors experienced a low quality of life for a lengthy period of time because of the sequelae of the syndrome, such as pulmonary function limitations and sustained neurocognitive deficiencies [4]. Sepsis is a critical infectious condition that can cause immune system responses and organ dysfunction [5]. Severe sepsis is known to cause fatal stages of disease development that involve lactic acidosis, oliguria, and ARDS [6].Many studies have attempted to elucidate how ARDS occurs, such as explaining how pulmonary edema fluid accumulates due to lung inflammation and increased alveolar endothelial and epithelial permeabilities [7–9]. Some studies have described how these pathways are disrupted in ARDS [10–15]. However, researchers have not yet elucidated the multifactorial mechanisms by which sepsis induces ARDS, nor have they distinguished the mechanism of sepsis and sepsis-induced ARDS, which could further explain how and why ARDS occur.

ARDS is characterized by a serious inflammatory reaction in the lung and leads to serious hypoxemia and poor pulmonary compliance in both children and adults [9, 16]. Despite development in the understanding of the pathogenesis of ARDS, the underlying mechanism still needs to be elucidated. Immune cells interact with and respond to lung infection, and their contribution to disease progression is critical for the development of effective management strategies [17]. In addition, surviving a severe respiratory infection is dependent on a careful balance between mounting an immune response that is sufficient to clear the infection and maintaining lung function despite immune-induced tissue damage [18]. Hence, studying the interrelationships between immune cells is important in the exploration of ARDS development.

At present, the single-cell biotechnologies provide an opportunity to identify new and rare cell types and their characteristics with unprecedented accuracy. Recent research has used single-cell RNA sequencing (scRNA-seq) to identify an early monocyte gene signature in ARDS [3]. In this study, to further elucidate the complex dynamic process of the immune system's response in ARDS, we construct the intercellular communication network of immune cells in ARDS based on scRNA-seq dataset (including three sepsis-induced ARDS patients and four sepsis-only patients). We then investigated the distinctive intercellular signals and the internal signaling in macrophages ofsepsis-induced ARDS. We believe that our study provides valuable clues for understanding the immune response mechanisms of ARDS.

## 2. Materials and Methods

### 2.1. Data Collection and Processing

Data were collected from GSE151263, including three sepsis-induced ARDS patients and four sepsis-only patients. The scRNA-seq and quality control were performed as described in [3]. Differentially expressed genes (DEGs) in the two groups were screened by “Seurat3” in R (p_adj < 0.05 and logFC > 0.4) and were drawn by “pheatmap” and “ggplot2” in R. The t-SNE was drawn by “Seurat” in R.

### 2.2. Cell Recognition

Table 1 lists the markers expressed specifically in immune cells, sourced from Garnett (version: 0.1.20) [19]. By observing the expression of these markers, we were able to recognize different immune cells in the patients.

### 2.3. Inferring Cell–Cell Communication

Intercellular signaling among different immune cell types of sepsis and sepsis-induced ARDS was calculated by CellCall [20], which is a toolkit for recognition of intercellular communication networks and internal regulatory signals by combining the expression of ligands/receptors with downstream transcription factor (TF) activities for certain ligand–receptor (LR) pairs. Genes that were expressed in less than 10% of the cells of a certain cell type were excluded in this study. For details, in CellCall package, we set the parameter of function CreateNichConObject() with min.feature = 3, scale.factor = 10^6^. Parameters p.adjust = 0.05 and probs = 0.9 in function TransCommuProfile(). Meanwhile, we have set the default parameter with function CreateSeuratObject() in Seurat package.

### 2.4. Disease Preference Analysis

Based on Zhang et al.'s work [21], we proposed the “disease preference” by calculating the index *D*:

(1) $$\begin{array}{c} D=\frac{\text{Observation}}{\text{Expected}}, \end{array}$$

where Observation means the number of specific immune cells in every patient (Table 2) and Expected equals (*M*/*S*)∗*N*, where *M* is the total number of specific immune cells in all the patients (e.g., *M* might be the total number of B cells in all seven patients). *S* is the total number of cells in all seven patients, and *N* is the total number of cells in one patient.

### 2.5. Statistical Analysis

The Louvain algorithm was applied for classified analysis (resolution = 0.5). Spearman correlation coefficient was used to calculate the relevance among different immune cells by assessing the mean value of cell markers present in different immune cells drawn by “psych” in R. Intercellular communication networks and downstream TF activities for certain LR pairs were observed, and a ridgeline plot was drawn using “CellCall” in R. Gene ontology functional enrichment analysis was performed on Metascape [22] with kappa similarity index.

## 3. Results

### 3.1. Intercellular Communications in Sepsis and Sepsis-Induced ARDS

**Figure 1:**
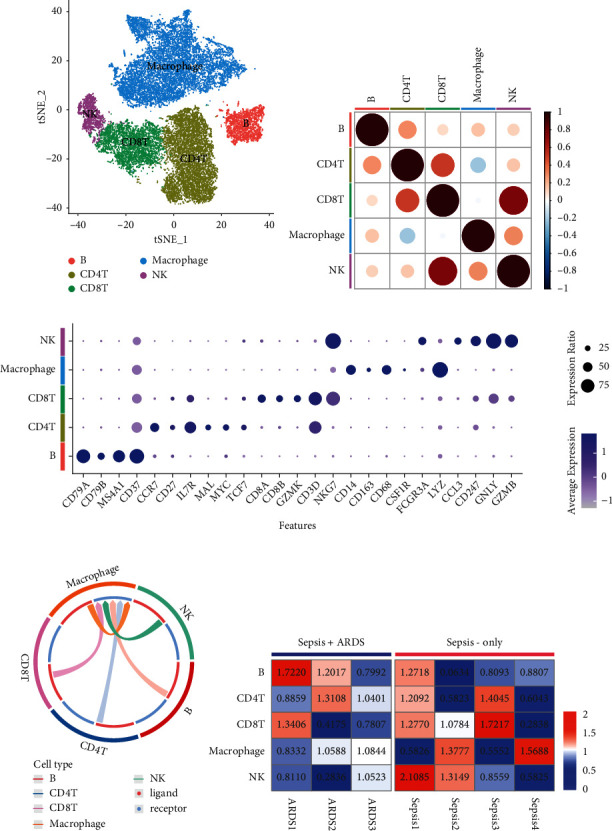
Characteristic of different immune cells. (a) t-SNE analysis for five clusters corresponding to five immune cells, respectively. (b) Correlation of immune cells. (c) Expression of markers related to classification of the five immune cells. (d) Intercellular signaling among different immune cell types. (e) Disease preference of macrophages and other immune cells for the same patient.

According to the Louvain algorithm, the scRNA-seq data were classified for five immune cell types (NK, B, CD4+ T, and CD8+ T cells) (Figure 1(a)).  Figure 1(c) illustrates the expression of markers to help classify the five cluster cells.  Figure 1(b) reveals little correlation among different immune cells, except NK cells and CD8+ T cells. Intercellular signaling among different immune cell types was analyzed by CellCall, and macrophages exhibited extensive communication with other immune cells (Figure 1(d)). Moreover, the disease preference results indicated that macrophages exhibit different trends from other cells in the same patient (Figure 1(e)).

### 3.2. DEG Analysis in Macrophage

**Figure 2:**
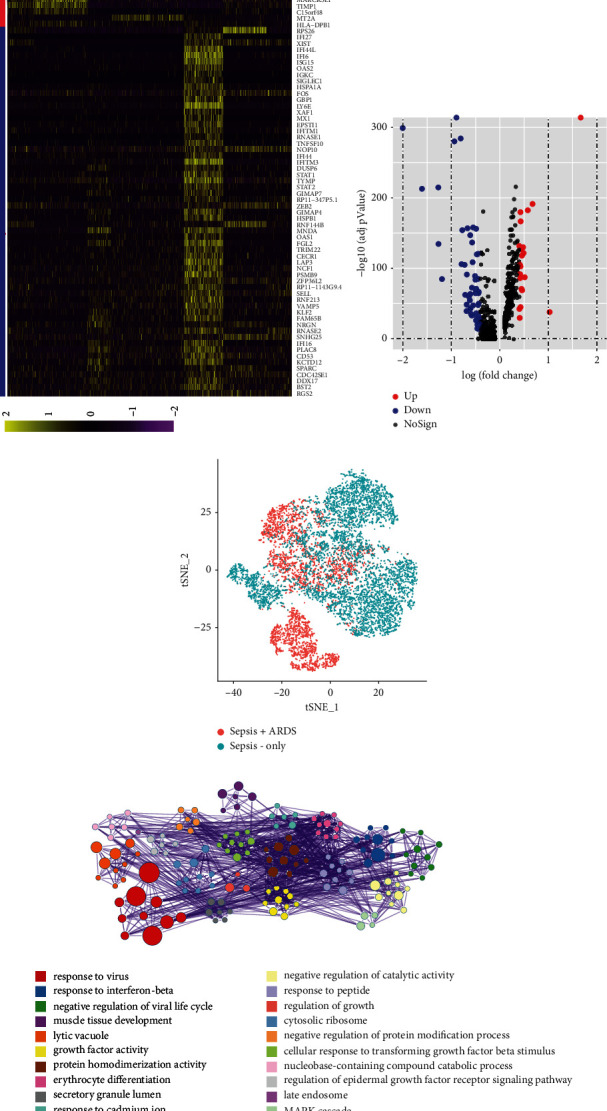
Differentially expressed gene (DEG) analysis and gene ontology functional enrichment analysis in macrophages. (a) Heatmap of DEGs in sepsis and sepsis-induced ARDS of macrophages. (b) Volcano plot displaying DEGs between the two groups in macrophages. (c) t-SNE analysis with top 20 major factors. (d) Gene ontology functional enrichment analysis with a total of 80 DEGs.

To compare the function of macrophages involved in the extensive intercellular communication in sepsis and sepsis-induced ARDS, DEG analysis was performed. We detected 21 genes expressed noticeably in sepsis-only patients, and 59 genes expressed noticeably in sepsis-induced ARDS patients (see Figures 2(a) and 2(b)). Among the genes, IFI44L is often expressed as a response to viral infections, which evokes extensive immunomodulation [23]. HLA-DQB1 is known as a major histocompatibility complex, showing connections to many immune cells and related to immune response [24, 25]. AREG, which encodes amphiregulin, was among the downregulated genes in sepsis-induced ARDS. Downregulation of AREG induces epithelial cell apoptosis in lipopolysaccharide-induced lung injury in mice [26].  Figure 2(c) reveals that the t-SNE results distinguish the two groups of samples, by performing PCA analysis with a total of 80 DEGs. Gene ontology functional enrichment analysis with a total of 80 DEGs is exhibited in Figure 2(d) and Table S1. Terms such as “response to virus,” “negative regulation of viral life cycle,” “growth factor activity,” and “response to interferon-beta” were related to immune response and regulation of a complex transcriptional response.

### 3.3. Differential Intercellular Communication Related to Macrophages

**Figure 3:**
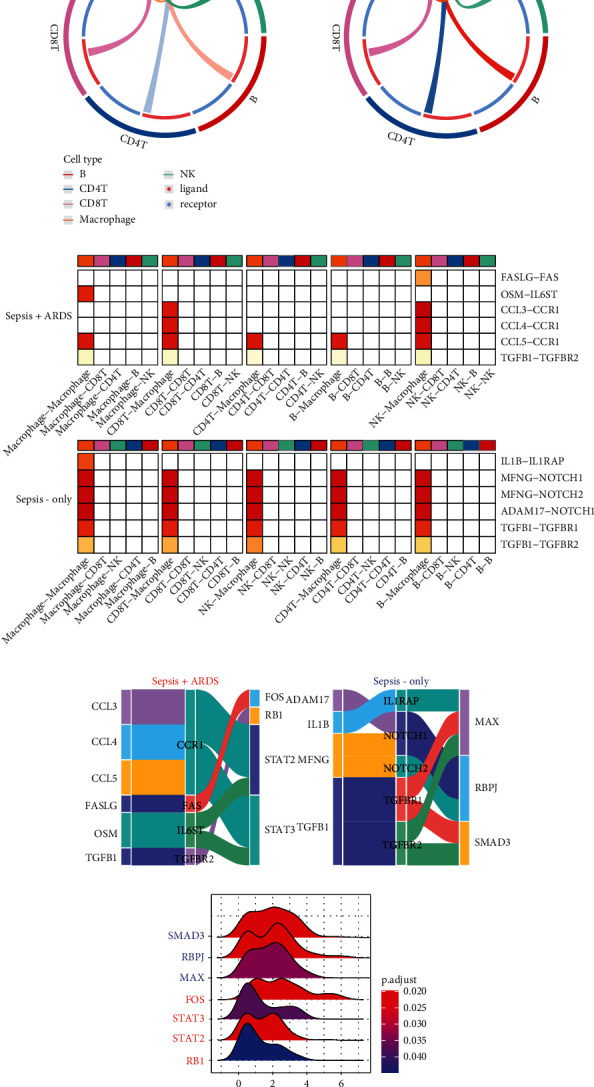
CellCall results and ridge plot of genes regulated by transcription factor (TF). (a) Intercellular signals from macrophage to other immune cells. (b) Ligand-receptor (LR) pairs between macrophage and other immune cells for two groups. (c) Downstream transcription factors (TFs) targeted by LR pairs in sepsis-induced ARDS and sepsis, and the width of each item means the frequencies of each pairs occurred among all the pairs, with the wider flow between two objects, the closer relation they have. (d) Ridgeline plot of genes regulated by TFs.

Intercellular signals from macrophages to other immune cells were noticeably higher in the sepsis-only patients compared to the sepsis-induced ARDS patients (Figure 3(a)). We list the LR pairs between macrophages and other immune cells for the two groups in Figure 3(b). Only one pair was observed to have the same ligand and receptor. Sepsis-induced ARDS patients displayed FASLG−FAS, which reportedly is related to increased protein permeability in the pulmonary alveoli [27], and CCL3/4/5−CCR1, which is related to the chemokines and proinflammatory cytokines that participate in and promote inflammatory responses related to macrophages [28, 29]. Sepsis-only patients displayed IL1B−IL1RAP, of which Interleukin-1-B (IL1B) is a proinflammatory cytokine that plays an important role in sepsis and affects the p38 MAPK and NF*κ*B signaling pathways [30, 31], and TGFB1−TGFBR1, of which transforming growth factor beta1 (TGF-beta1) gene single-nucleotide polymorphisms and plasma TGF-beta1 levels were thought to be associated with susceptibility to sepsis [32]. Furthermore, we analyzed the downstream TFs targeted by LR pairs (Figure 3(c)) and found that different TFs, including SMAD3, RBPJ, and MAX, were targeted in sepsis-only patients, while FOS, STAT3, STAT2, and RB1 were targeted in sepsis-induced patients. Furthermore, most genes regulated by TFs were differentially expressed (Figure 3(d)), which could prove the downstream TFs were activated. However, the genes regulated by STAT3 were low expression, which may indicate that STAT3 was inhibited.

## 4. Discussion

ARDS is a syndrome of acute respiratory failure caused by noncardiogenic pulmonary edema [11], resulting in an excessive inflammatory and immune response [33]. In this study, we analyzed intercellular communication of immune cell types with scRNA-seq data to identify the immune cell and downstream TFs that play a vital role in sepsis and sepsis-induced ARDS. Our results indicate that macrophages have the most extensive communication with other immune cells, such as NK, B, CD4+ T, and CD8+ T cells. We further screened the DEGs in macrophages between sepsis and sepsis-induced ARDS. The DEGs were enriched in gene ontology terms, such as “response to virus,” “negative regulation of viral life cycle,” and “regulation of epidermal growth factor receptor signaling pathway,” which related to immune response. We compared the LR pairs between macrophages and other immune cells in the two groups. The LR pairs in the sepsis-induced ARDS group, including FASLG−FAS, OSM−IL6ST, and CCL3/4/5−CCR1, were different from pairs in the sepsis-only group. The chemokine–chemokine receptors (CCL3/4/5−CCR1) are known to be involved in the promotion inflammatory response by macrophages [28, 29, 34]. FASLG−FAS were involved in protein permeability in the pulmonary alveoli [27]. Moreover, the downstream TFs targeted by the LR pairs in sepsis-induced ARDS are FOS, RB1, STAT2, and STAT3. In vivo, aberrant expression of Stat3 has been associated with immune tolerance [35], acute-phase response [36], and septic shock [37]. These observations suggest that Stat3 may play an important role during inflammation [38]. Other studies have demonstrated the relationship between STAT3 and macrophages as well as macrophage phenotype shift.

Some researchers have confirmed that macrophages play a dual role of proinflammation and anti-inflammation based on the microenvironment in different pathological stages. In the acute phase of ALI/ARDS, resident alveolar macrophages, typically expressing the alternatively activated phenotype (M2), shift into the classically activated phenotype (M1) and release various potent proinflammatory mediators. In the later phase, the M1 phenotype of the activated resident and recruited macrophages shifts back to the M2 phenotype for eliminating apoptotic cells and participating in fibrosis [39–45]. If the process of shifting back to M2 is blocked, then the severe inflammatory reactions will persist and ARDS will not proceed to the next stage. Yin et al. [46] have found inhibition of the IL-6/STAT3 signaling pathway can induce the polarization of M1 macrophages and suppress the polarization of M2 macrophages. The inhibition of the IL-6/STAT3 signaling pathway can turn macrophages into M1 type, which is in line with our results that STAT3 was inhibited. Chen et al. [44] provide evidence that the transcription factor STAT3 can promote the transcription of lnc-M2 and facilitate the process of M2 macrophage differentiation via the PKA/CREB pathway. In a breast cancer study, Griess et al. [45] found that the inhibition of M2 marker genes was partly mediated through a decrease in Stat3 activation during IL4-induced M2 polarization [47]. Furthermore, the expression of STAT3 was successfully reduced after STAT3 knockdown, resulting in an increase in inflammation and M1 macrophages and a decrease in the proportion of M2 macrophages [48].

## 5. Conclusion

In this study, we inferred the intercellular communication of immune cells and, with scRNA-seq data analysis, found that macrophages play a vital role in sepsis and sepsis-induced ARDS. Meanwhile, downstream TFs, including STAT3, were found to play a vital role in the process of macrophage M1/M2 polarization. Determining in what case the upstream LR pairs combined to activate STAT3 requires further research. In summary, this study provides a comprehensive view of the intercellular communication landscape between sepsis and sepsis-induced ARDS and identifies key intercellular communications and TFs involved in sepsis-induced ARDS. We believe that our study provides valuable clues for understanding the immune response mechanisms of ARDS.

## Figures and Tables

**Table 1:** Markers of different immune cells.

Cell type	Subtype	Marker
B cell		CD79A, CD79B, MS4A1, CD37
CD4+ T cell	CD4+ memory T cell	CCR7, CD27, IL7R
	Naive CD4+ T cell	CCR7, IL7R, MAL, MYC, TCF7
CD8 T cell		CD8A, CD8B, GZMK, CD3D, NKG7
Macrophage cell		CD14, CD163, CD68, CSF1R, FCGR3A, LYZ
NK cell		CCL3, CD247, GNLY, GZMB, NKG7

**Table 2:** Number of specific immune cells in each patient.

	Sepsis 1	Sepsis 2	Sepsis 3	Sepsis 4	ARDS 1	ARDS 2	ARDS 3
B	419	18	248	298	594	357	134
CD4T	1245	517	1345	639	955	1217	545
CD8T	736	536	923	168	809	217	229
Macrophage	969	1976	859	2680	1451	1588	918
NK	437	235	165	124	176	53	111
Sum	3806	3282	3540	3909	3985	3432	1937

## Data Availability

The data used to support the findings of this study are included within the article.

## Supplementary Materials

Supplementary Materials Gene ontology functional enrichment analysis with a total of 80 DEGs is exhibited in Table S1.
