# Supplementary material for: Investigating the Intercellular Communication Network of Immune Cell in Acute Respiratory Distress Syndrome with Sepsis
Source: Comput Math Methods Med. 2022 Feb 16;2022:4586648. doi: 10.1155/2022/4586648 (PMC8866031; doi:10.1155/2022/4586648)
Supplement: Supplementary Materials — Gene ontology functional enrichment analysis with a total of 80 DEGs is exhibited in Table S1. [file 4586648.f1.docx]

| **GroupID** | **Term** | **Description** | **LogP** | **Log(q-value)** | **Symbols** |
| --- | --- | --- | --- | --- | --- |
| 1_Summary | GO:0009615 | response to virus | -21.3395 | -17.235 | BST2,IFI6,GBP1,HSPB1,IFI16,IFI27,MX1,OAS1,OAS2,RNASE1,RNASE2,STAT1,STAT2,IFITM1,ISG15,TRIM22,IFITM3,DDX17,IFI44,FGL2,IFI44L,LY6E,HSPA1A,SIGLEC1,OSM,MNDA,RIPOR2,NCF1,SOCS3,FOS,IGKC,SPARC,PLAC8,C15orf48 |
| 1_Member | GO:0009615 | response to virus | -21.3395 | -17.235 | BST2,IFI6,GBP1,HSPB1,IFI16,IFI27,MX1,OAS1,OAS2,RNASE1,RNASE2,STAT1,STAT2,IFITM1,ISG15,TRIM22,IFITM3,DDX17,IFI44,FGL2,IFI44L |
| 1_Member | GO:0051607 | defense response to virus | -21.1101 | -17.235 | BST2,IFI6,GBP1,IFI16,IFI27,MX1,OAS1,OAS2,RNASE1,RNASE2,STAT1,STAT2,IFITM1,ISG15,TRIM22,IFITM3,DDX17,FGL2,IFI44L |
| 1_Member | GO:0140546 | defense response to symbiont | -21.1101 | -17.235 | BST2,IFI6,GBP1,IFI16,IFI27,MX1,OAS1,OAS2,RNASE1,RNASE2,STAT1,STAT2,IFITM1,ISG15,TRIM22,IFITM3,DDX17,FGL2,IFI44L |
| 1_Member | GO:0034340 | response to type I interferon | -13.2451 | -9.592 | IFI27,MX1,OAS1,OAS2,STAT1,STAT2,IFITM1,ISG15,IFITM3 |
| 1_Member | GO:0048525 | negative regulation of viral process | -13.0564 | -9.483 | BST2,IFI16,LY6E,MX1,OAS1,OAS2,STAT1,IFITM1,ISG15,IFITM3 |
| 1_Member | GO:0050792 | regulation of viral process | -11.9851 | -8.483 | BST2,IFI16,LY6E,MX1,OAS1,OAS2,STAT1,IFITM1,ISG15,TRIM22,IFITM3 |
| 1_Member | GO:0060337 | type I interferon signaling pathway | -11.9315 | -8.483 | IFI27,OAS1,OAS2,STAT1,STAT2,IFITM1,ISG15,IFITM3 |
| 1_Member | GO:0071357 | cellular response to type I interferon | -11.7868 | -8.389 | IFI27,OAS1,OAS2,STAT1,STAT2,IFITM1,ISG15,IFITM3 |
| 1_Member | GO:0045071 | negative regulation of viral genome replication | -11.5147 | -8.163 | BST2,IFI16,MX1,OAS1,OAS2,IFITM1,ISG15,IFITM3 |
| 1_Member | GO:0019058 | viral life cycle | -11.3623 | -8.052 | BST2,HSPA1A,IFI16,IFI27,LY6E,MX1,OAS1,OAS2,SIGLEC1,IFITM1,ISG15,TRIM22,IFITM3 |
| 1_Member | GO:0016032 | viral process | -11.0847 | -7.812 | BST2,HSPA1A,IFI16,IFI27,LY6E,MX1,OAS1,OAS2,SIGLEC1,STAT1,IFITM1,ISG15,TRIM22,IFITM3 |
| 1_Member | GO:1903900 | regulation of viral life cycle | -10.9601 | -7.722 | BST2,IFI16,LY6E,MX1,OAS1,OAS2,IFITM1,ISG15,TRIM22,IFITM3 |
| 1_Member | GO:0045069 | regulation of viral genome replication | -10.0127 | -6.807 | BST2,IFI16,MX1,OAS1,OAS2,IFITM1,ISG15,IFITM3 |
| 1_Member | GO:0019079 | viral genome replication | -9.96885 | -6.793 | BST2,IFI16,IFI27,MX1,OAS1,OAS2,IFITM1,ISG15,IFITM3 |
| 1_Member | GO:0070106 | interleukin-27-mediated signaling pathway | -8.72265 | -5.575 | MX1,OAS1,OAS2,STAT1 |
| 1_Member | GO:0019221 | cytokine-mediated signaling pathway | -7.13081 | -4.009 | HSPA1A,IFI27,MX1,OAS1,OAS2,OSM,STAT1,STAT2,IFITM1,ISG15,IFITM3 |
| 1_Member | GO:0060700 | regulation of ribonuclease activity | -5.77038 | -2.765 | HSPA1A,OAS1,OAS2 |
| 1_Member | GO:0002683 | negative regulation of immune system process | -5.50055 | -2.544 | BST2,GBP1,IFI16,MNDA,OAS1,STAT2,ISG15,RIPOR2,FGL2 |
| 1_Member | GO:0002831 | regulation of response to biotic stimulus | -5.45432 | -2.517 | IFI16,MNDA,OAS1,STAT1,STAT2,ISG15,FGL2,NCF1 |
| 1_Member | GO:0031347 | regulation of defense response | -5.05795 | -2.220 | IFI16,MNDA,OAS1,OSM,STAT1,STAT2,SOCS3,ISG15,FGL2,NCF1 |
| 1_Member | GO:0002832 | negative regulation of response to biotic stimulus | -4.89376 | -2.144 | IFI16,OAS1,STAT2,ISG15,FGL2 |
| 1_Member | GO:0032481 | positive regulation of type I interferon production | -4.68562 | -2.006 | OAS1,OAS2,STAT1,ISG15 |
| 1_Member | GO:0060339 | negative regulation of type I interferon-mediated signaling pathway | -4.6473 | -1.994 | OAS1,STAT2,ISG15 |
| 1_Member | GO:0032069 | regulation of nuclease activity | -4.51842 | -1.899 | HSPA1A,OAS1,OAS2 |
| 1_Member | GO:0045088 | regulation of innate immune response | -4.48897 | -1.893 | IFI16,MNDA,OAS1,STAT2,ISG15,NCF1 |
| 1_Member | GO:0009617 | response to bacterium | -4.3909 | -1.824 | FOS,IGKC,OAS1,OAS2,RNASE2,SPARC,ISG15,IFI44,PLAC8,C15orf48 |
| 1_Member | GO:0045824 | negative regulation of innate immune response | -4.3376 | -1.790 | IFI16,OAS1,STAT2,ISG15 |
| 1_Member | GO:0031348 | negative regulation of defense response | -4.08325 | -1.640 | IFI16,OAS1,STAT2,SOCS3,ISG15,FGL2 |
| 1_Member | GO:0060338 | regulation of type I interferon-mediated signaling pathway | -3.86437 | -1.497 | OAS1,STAT2,ISG15 |
| 1_Member | GO:0032479 | regulation of type I interferon production | -3.84403 | -1.488 | OAS1,OAS2,STAT1,ISG15 |
| 1_Member | GO:0032606 | type I interferon production | -3.84403 | -1.488 | OAS1,OAS2,STAT1,ISG15 |
| 1_Member | GO:0032728 | positive regulation of interferon-beta production | -3.75976 | -1.429 | OAS1,OAS2,ISG15 |
| 1_Member | GO:0050777 | negative regulation of immune response | -3.68403 | -1.374 | IFI16,OAS1,STAT2,ISG15,FGL2 |
| 1_Member | GO:0042742 | defense response to bacterium | -3.343 | -1.131 | IGKC,OAS1,OAS2,RNASE2,ISG15,PLAC8 |
| 1_Member | GO:0032608 | interferon-beta production | -3.29256 | -1.093 | OAS1,OAS2,ISG15 |
| 1_Member | GO:0032648 | regulation of interferon-beta production | -3.29256 | -1.093 | OAS1,OAS2,ISG15 |
| 1_Member | GO:0001959 | regulation of cytokine-mediated signaling pathway | -3.0902 | -0.949 | HSPA1A,OAS1,STAT2,ISG15 |
| 1_Member | GO:0060759 | regulation of response to cytokine stimulus | -2.96607 | -0.862 | HSPA1A,OAS1,STAT2,ISG15 |
| 1_Member | GO:0032102 | negative regulation of response to external stimulus | -2.96234 | -0.861 | IFI16,OAS1,STAT2,SOCS3,ISG15,FGL2 |
| 1_Member | GO:0001960 | negative regulation of cytokine-mediated signaling pathway | -2.82586 | -0.768 | OAS1,STAT2,ISG15 |
| 1_Member | GO:0060761 | negative regulation of response to cytokine stimulus | -2.75117 | -0.719 | OAS1,STAT2,ISG15 |
| 1_Member | GO:0032760 | positive regulation of tumor necrosis factor production | -2.54021 | -0.558 | HSPB1,OAS1,OAS2 |
| 1_Member | GO:1903557 | positive regulation of tumor necrosis factor superfamily cytokine production | -2.49304 | -0.527 | HSPB1,OAS1,OAS2 |
| 2_Summary | GO:0035456 | response to interferon-beta | -13.6221 | -9.872 | BST2,IFI16,MNDA,OAS1,STAT1,IFITM1,IFITM3,XAF1,GBP1,HLA-DPB1,HSPA1A,HSPB1,OAS2,OSM,ISG15,KLF2,VEGFA,RIPOR2 |
| 2_Member | GO:0035456 | response to interferon-beta | -13.6221 | -9.872 | BST2,IFI16,MNDA,OAS1,STAT1,IFITM1,IFITM3,XAF1 |
| 2_Member | GO:0001817 | regulation of cytokine production | -6.54905 | -3.452 | BST2,GBP1,HLA-DPB1,HSPA1A,HSPB1,IFI16,MNDA,OAS1,OAS2,OSM,STAT1,ISG15,KLF2 |
| 2_Member | GO:0035458 | cellular response to interferon-beta | -6.25588 | -3.183 | IFI16,MNDA,OAS1,STAT1 |
| 2_Member | GO:0001819 | positive regulation of cytokine production | -6.18245 | -3.151 | HLA-DPB1,HSPA1A,HSPB1,IFI16,MNDA,OAS1,OAS2,OSM,STAT1,ISG15 |
| 2_Member | GO:0032731 | positive regulation of interleukin-1 beta production | -3.16281 | -0.998 | HSPB1,IFI16,MNDA |
| 2_Member | GO:0032732 | positive regulation of interleukin-1 production | -2.95626 | -0.860 | HSPB1,IFI16,MNDA |
| 2_Member | GO:0032103 | positive regulation of response to external stimulus | -2.92032 | -0.836 | HSPB1,IFI16,MNDA,OSM,VEGFA,RIPOR2 |
| 2_Member | GO:0032611 | interleukin-1 beta production | -2.43672 | -0.490 | HSPB1,IFI16,MNDA |
| 2_Member | GO:0032651 | regulation of interleukin-1 beta production | -2.43672 | -0.490 | HSPB1,IFI16,MNDA |
| 2_Member | GO:0032612 | interleukin-1 production | -2.25469 | -0.350 | HSPB1,IFI16,MNDA |
| 2_Member | GO:0032652 | regulation of interleukin-1 production | -2.25469 | -0.350 | HSPB1,IFI16,MNDA |
| 2_Member | GO:0050921 | positive regulation of chemotaxis | -2.1482 | -0.265 | HSPB1,VEGFA,RIPOR2 |
| 3_Summary | GO:1903901 | negative regulation of viral life cycle | -6.18107 | -3.151 | BST2,LY6E,IFITM1,IFITM3,HSPA1A,SIGLEC1,TRIM22,IFI27,GBP1,STAT1 |
| 3_Member | GO:1903901 | negative regulation of viral life cycle | -6.18107 | -3.151 | BST2,LY6E,IFITM1,IFITM3 |
| 3_Member | GO:0046718 | viral entry into host cell | -5.49751 | -2.544 | HSPA1A,LY6E,SIGLEC1,IFITM1,TRIM22,IFITM3 |
| 3_Member | GO:0044409 | entry into host | -5.37919 | -2.459 | HSPA1A,LY6E,SIGLEC1,IFITM1,TRIM22,IFITM3 |
| 3_Member | GO:0046596 | regulation of viral entry into host cell | -5.24973 | -2.375 | LY6E,IFITM1,TRIM22,IFITM3 |
| 3_Member | GO:0052126 | movement in host environment | -5.01401 | -2.194 | HSPA1A,LY6E,SIGLEC1,IFITM1,TRIM22,IFITM3 |
| 3_Member | GO:0052372 | modulation by symbiont of entry into host | -4.97898 | -2.173 | LY6E,IFITM1,TRIM22,IFITM3 |
| 3_Member | GO:0044403 | biological process involved in symbiotic interaction | -4.75315 | -2.043 | HSPA1A,IFI27,LY6E,SIGLEC1,IFITM1,TRIM22,IFITM3 |
| 3_Member | GO:0051701 | biological process involved in interaction with host | -4.65072 | -1.994 | HSPA1A,LY6E,SIGLEC1,IFITM1,TRIM22,IFITM3 |
| 3_Member | GO:0035455 | response to interferon-alpha | -4.58122 | -1.945 | BST2,IFITM1,IFITM3 |
| 3_Member | GO:0046597 | negative regulation of viral entry into host cell | -4.58122 | -1.945 | LY6E,IFITM1,IFITM3 |
| 3_Member | GO:0034341 | response to interferon-gamma | -4.33601 | -1.790 | BST2,GBP1,STAT1,IFITM1,IFITM3 |
| 3_Member | GO:0043903 | regulation of biological process involved in symbiotic interaction | -4.31369 | -1.781 | LY6E,IFITM1,TRIM22,IFITM3 |
| 4_Summary | GO:0060537 | muscle tissue development | -5.75505 | -2.765 | ENG,FOS,LY6E,RGS2,VEGFA,RIPOR2,DDX17,VAMP5,TIPARP,CD53 |
| 4_Member | GO:0060537 | muscle tissue development | -5.75505 | -2.765 | ENG,FOS,LY6E,RGS2,VEGFA,RIPOR2,DDX17,VAMP5,TIPARP |
| 4_Member | GO:0014706 | striated muscle tissue development | -4.94526 | -2.173 | ENG,FOS,LY6E,RGS2,VEGFA,RIPOR2,DDX17,VAMP5 |
| 4_Member | GO:0061061 | muscle structure development | -4.18161 | -1.681 | CD53,ENG,FOS,LY6E,RGS2,VEGFA,RIPOR2,DDX17,VAMP5 |
| 4_Member | GO:0007517 | muscle organ development | -3.5257 | -1.248 | ENG,FOS,LY6E,RIPOR2,DDX17,VAMP5 |
| 4_Member | GO:0007519 | skeletal muscle tissue development | -3.04765 | -0.924 | FOS,RIPOR2,DDX17,VAMP5 |
| 4_Member | GO:0060538 | skeletal muscle organ development | -2.93661 | -0.850 | FOS,RIPOR2,DDX17,VAMP5 |
| 5_Summary | GO:0000323 | lytic vacuole | -5.28217 | -2.393 | BST2,HLA-DPB1,HLA-DQB1,HLA-DRB5,MNDA,RNASE2,IFITM1,IFITM3,PLAC8,ADA2,NCF1,ZFP36L2,MAP3K8,IGKC,NFIL3,RIPOR2,FGL2,AREG,NRGN,SELL,VEGFA,GBP1,HSPA1A,OAS1,SPARC,IFI16,ENG,RGS2,RPS26,VAMP5,TMEM70,LAP3 |
| 5_Member | GO:0000323 | lytic vacuole | -5.28217 | -2.393 | BST2,HLA-DPB1,HLA-DQB1,HLA-DRB5,MNDA,RNASE2,IFITM1,IFITM3,PLAC8,ADA2,NCF1 |
| 5_Member | GO:0005764 | lysosome | -5.28217 | -2.393 | BST2,HLA-DPB1,HLA-DQB1,HLA-DRB5,MNDA,RNASE2,IFITM1,IFITM3,PLAC8,ADA2,NCF1 |
| 5_Member | GO:0046649 | lymphocyte activation | -5.05306 | -2.220 | ZFP36L2,BST2,MAP3K8,HLA-DPB1,HLA-DQB1,HLA-DRB5,IGKC,MNDA,NFIL3,RIPOR2,FGL2 |
| 5_Member | GO:0002399 | MHC class II protein complex assembly | -4.95255 | -2.173 | HLA-DPB1,HLA-DQB1,HLA-DRB5 |
| 5_Member | GO:0002503 | peptide antigen assembly with MHC class II protein complex | -4.95255 | -2.173 | HLA-DPB1,HLA-DQB1,HLA-DRB5 |
| 5_Member | GO:0051249 | regulation of lymphocyte activation | -4.89578 | -2.144 | ZFP36L2,MAP3K8,HLA-DPB1,HLA-DQB1,HLA-DRB5,IGKC,MNDA,RIPOR2,FGL2 |
| 5_Member | GO:0042613 | MHC class II protein complex | -4.86909 | -2.130 | HLA-DPB1,HLA-DQB1,HLA-DRB5 |
| 5_Member | GO:0002501 | peptide antigen assembly with MHC protein complex | -4.79078 | -2.062 | HLA-DPB1,HLA-DQB1,HLA-DRB5 |
| 5_Member | GO:0030665 | clathrin-coated vesicle membrane | -4.74322 | -2.043 | AREG,HLA-DPB1,HLA-DQB1,HLA-DRB5,NRGN |
| 5_Member | GO:0002396 | MHC protein complex assembly | -4.71701 | -2.028 | HLA-DPB1,HLA-DQB1,HLA-DRB5 |
| 5_Member | GO:0012507 | ER to Golgi transport vesicle membrane | -4.57039 | -1.943 | AREG,HLA-DPB1,HLA-DQB1,HLA-DRB5 |
| 5_Member | GO:0002694 | regulation of leukocyte activation | -4.3428 | -1.790 | ZFP36L2,MAP3K8,HLA-DPB1,HLA-DQB1,HLA-DRB5,IGKC,MNDA,RIPOR2,FGL2 |
| 5_Member | GO:0030669 | clathrin-coated endocytic vesicle membrane | -4.31369 | -1.781 | AREG,HLA-DPB1,HLA-DQB1,HLA-DRB5 |
| 5_Member | GO:0042611 | MHC protein complex | -4.29443 | -1.769 | HLA-DPB1,HLA-DQB1,HLA-DRB5 |
| 5_Member | GO:0023026 | MHC class II protein complex binding | -4.24414 | -1.731 | HLA-DPB1,HLA-DQB1,HLA-DRB5 |
| 5_Member | GO:0071556 | integral component of lumenal side of endoplasmic reticulum membrane | -4.14923 | -1.661 | HLA-DPB1,HLA-DQB1,HLA-DRB5 |
| 5_Member | GO:0098553 | lumenal side of endoplasmic reticulum membrane | -4.14923 | -1.661 | HLA-DPB1,HLA-DQB1,HLA-DRB5 |
| 5_Member | GO:0007159 | leukocyte cell-cell adhesion | -4.11316 | -1.647 | MAP3K8,HLA-DPB1,HLA-DQB1,HLA-DRB5,SELL,RIPOR2,FGL2 |
| 5_Member | GO:0050865 | regulation of cell activation | -4.10945 | -1.647 | ZFP36L2,MAP3K8,HLA-DPB1,HLA-DQB1,HLA-DRB5,IGKC,MNDA,RIPOR2,FGL2 |
| 5_Member | GO:0019886 | antigen processing and presentation of exogenous peptide antigen via MHC class II | -4.10434 | -1.647 | HLA-DPB1,HLA-DQB1,HLA-DRB5 |
| 5_Member | GO:0002495 | antigen processing and presentation of peptide antigen via MHC class II | -3.93934 | -1.527 | HLA-DPB1,HLA-DQB1,HLA-DRB5 |
| 5_Member | GO:0045334 | clathrin-coated endocytic vesicle | -3.91635 | -1.514 | AREG,HLA-DPB1,HLA-DQB1,HLA-DRB5 |
| 5_Member | GO:0002504 | antigen processing and presentation of peptide or polysaccharide antigen via MHC class II | -3.86437 | -1.497 | HLA-DPB1,HLA-DQB1,HLA-DRB5 |
| 5_Member | GO:0023023 | MHC protein complex binding | -3.86437 | -1.497 | HLA-DPB1,HLA-DQB1,HLA-DRB5 |
| 5_Member | GO:0098576 | lumenal side of membrane | -3.86437 | -1.497 | HLA-DPB1,HLA-DQB1,HLA-DRB5 |
| 5_Member | GO:0030134 | COPII-coated ER to Golgi transport vesicle | -3.8618 | -1.497 | AREG,HLA-DPB1,HLA-DQB1,HLA-DRB5 |
| 5_Member | GO:0030662 | coated vesicle membrane | -3.82415 | -1.477 | AREG,HLA-DPB1,HLA-DQB1,HLA-DRB5,NRGN |
| 5_Member | GO:0002478 | antigen processing and presentation of exogenous peptide antigen | -3.79366 | -1.459 | HLA-DPB1,HLA-DQB1,HLA-DRB5 |
| 5_Member | GO:0042605 | peptide antigen binding | -3.79366 | -1.459 | HLA-DPB1,HLA-DQB1,HLA-DRB5 |
| 5_Member | GO:0019882 | antigen processing and presentation | -3.66078 | -1.358 | HLA-DPB1,HLA-DQB1,HLA-DRB5,FGL2 |
| 5_Member | GO:0030136 | clathrin-coated vesicle | -3.65316 | -1.354 | AREG,HLA-DPB1,HLA-DQB1,HLA-DRB5,NRGN |
| 5_Member | GO:0022407 | regulation of cell-cell adhesion | -3.5975 | -1.310 | MAP3K8,HLA-DPB1,HLA-DQB1,HLA-DRB5,VEGFA,RIPOR2,FGL2 |
| 5_Member | GO:0019884 | antigen processing and presentation of exogenous antigen | -3.51766 | -1.248 | HLA-DPB1,HLA-DQB1,HLA-DRB5 |
| 5_Member | GO:0030658 | transport vesicle membrane | -3.50622 | -1.243 | AREG,HLA-DPB1,HLA-DQB1,HLA-DRB5,NRGN |
| 5_Member | GO:0050863 | regulation of T cell activation | -3.50456 | -1.243 | MAP3K8,HLA-DPB1,HLA-DQB1,HLA-DRB5,RIPOR2,FGL2 |
| 5_Member | GO:0002764 | immune response-regulating signaling pathway | -3.47247 | -1.217 | GBP1,HLA-DPB1,HLA-DQB1,HSPA1A,IGKC,MNDA,OAS1 |
| 5_Member | GO:0030139 | endocytic vesicle | -3.46294 | -1.215 | AREG,HLA-DPB1,HLA-DQB1,HLA-DRB5,SPARC,NCF1 |
| 5_Member | GO:1903037 | regulation of leukocyte cell-cell adhesion | -3.46294 | -1.215 | MAP3K8,HLA-DPB1,HLA-DQB1,HLA-DRB5,RIPOR2,FGL2 |
| 5_Member | GO:0042110 | T cell activation | -3.40713 | -1.171 | ZFP36L2,MAP3K8,HLA-DPB1,HLA-DQB1,HLA-DRB5,RIPOR2,FGL2 |
| 5_Member | GO:0050851 | antigen receptor-mediated signaling pathway | -3.26052 | -1.067 | GBP1,HLA-DPB1,HLA-DQB1,IGKC,MNDA |
| 5_Member | GO:0002253 | activation of immune response | -3.20563 | -1.021 | GBP1,HLA-DPB1,HLA-DQB1,IFI16,IGKC,MNDA |
| 5_Member | GO:0048002 | antigen processing and presentation of peptide antigen | -3.16281 | -0.998 | HLA-DPB1,HLA-DQB1,HLA-DRB5 |
| 5_Member | GO:0005765 | lysosomal membrane | -3.0773 | -0.943 | BST2,HLA-DPB1,HLA-DQB1,HLA-DRB5,IFITM1,IFITM3 |
| 5_Member | GO:0098852 | lytic vacuole membrane | -3.0773 | -0.943 | BST2,HLA-DPB1,HLA-DQB1,HLA-DRB5,IFITM1,IFITM3 |
| 5_Member | GO:0002381 | immunoglobulin production involved in immunoglobulin-mediated immune response | -3.00913 | -0.895 | HLA-DPB1,HLA-DQB1,HLA-DRB5 |
| 5_Member | GO:0030155 | regulation of cell adhesion | -2.95187 | -0.860 | MAP3K8,GBP1,HLA-DPB1,HLA-DQB1,HLA-DRB5,VEGFA,RIPOR2,FGL2 |
| 5_Member | GO:0002429 | immune response-activating cell surface receptor signaling pathway | -2.88636 | -0.809 | GBP1,HLA-DPB1,HLA-DQB1,IGKC,MNDA |
| 5_Member | GO:0002757 | immune response-activating signal transduction | -2.88636 | -0.809 | GBP1,HLA-DPB1,HLA-DQB1,IGKC,MNDA |
| 5_Member | GO:0003823 | antigen binding | -2.85166 | -0.783 | HLA-DPB1,HLA-DQB1,HLA-DRB5,IGKC |
| 5_Member | GO:0030135 | coated vesicle | -2.82813 | -0.768 | AREG,HLA-DPB1,HLA-DQB1,HLA-DRB5,NRGN |
| 5_Member | GO:0005774 | vacuolar membrane | -2.81925 | -0.764 | BST2,HLA-DPB1,HLA-DQB1,HLA-DRB5,IFITM1,IFITM3 |
| 5_Member | GO:0002440 | production of molecular mediator of immune response | -2.77188 | -0.731 | BST2,HLA-DPB1,HLA-DQB1,HLA-DRB5,IGKC |
| 5_Member | GO:0098552 | side of membrane | -2.74066 | -0.715 | ENG,HLA-DPB1,HLA-DQB1,HLA-DRB5,IGKC,RGS2,RPS26 |
| 5_Member | GO:0002768 | immune response-regulating cell surface receptor signaling pathway | -2.73542 | -0.712 | GBP1,HLA-DPB1,HLA-DQB1,IGKC,MNDA |
| 5_Member | GO:0030666 | endocytic vesicle membrane | -2.68731 | -0.676 | AREG,HLA-DPB1,HLA-DQB1,HLA-DRB5 |
| 5_Member | GO:0002252 | immune effector process | -2.64192 | -0.636 | BST2,HLA-DPB1,HLA-DQB1,HLA-DRB5,IGKC,FGL2,NCF1 |
| 5_Member | GO:0016064 | immunoglobulin mediated immune response | -2.57737 | -0.579 | HLA-DPB1,HLA-DQB1,HLA-DRB5,IGKC |
| 5_Member | GO:0032588 | trans-Golgi network membrane | -2.57692 | -0.579 | HLA-DPB1,HLA-DQB1,HLA-DRB5 |
| 5_Member | GO:0019724 | B cell mediated immunity | -2.5549 | -0.563 | HLA-DPB1,HLA-DQB1,HLA-DRB5,IGKC |
| 5_Member | GO:0050870 | positive regulation of T cell activation | -2.51103 | -0.539 | MAP3K8,HLA-DPB1,HLA-DQB1,HLA-DRB5 |
| 5_Member | GO:0002377 | immunoglobulin production | -2.50385 | -0.534 | HLA-DPB1,HLA-DQB1,HLA-DRB5,IGKC |
| 5_Member | GO:0051251 | positive regulation of lymphocyte activation | -2.47522 | -0.520 | MAP3K8,HLA-DPB1,HLA-DQB1,HLA-DRB5,IGKC |
| 5_Member | GO:1903039 | positive regulation of leukocyte cell-cell adhesion | -2.3548 | -0.423 | MAP3K8,HLA-DPB1,HLA-DQB1,HLA-DRB5 |
| 5_Member | GO:0031301 | integral component of organelle membrane | -2.35258 | -0.422 | HLA-DPB1,HLA-DQB1,HLA-DRB5,VAMP5,TMEM70 |
| 5_Member | GO:0050852 | T cell receptor signaling pathway | -2.3121 | -0.390 | GBP1,HLA-DPB1,HLA-DQB1 |
| 5_Member | GO:0050778 | positive regulation of immune response | -2.27688 | -0.364 | GBP1,HLA-DPB1,HLA-DQB1,IFI16,IGKC,MNDA |
| 5_Member | GO:0002696 | positive regulation of leukocyte activation | -2.25227 | -0.349 | MAP3K8,HLA-DPB1,HLA-DQB1,HLA-DRB5,IGKC |
| 5_Member | GO:0030133 | transport vesicle | -2.23473 | -0.333 | AREG,HLA-DPB1,HLA-DQB1,HLA-DRB5,NRGN |
| 5_Member | GO:0005802 | trans-Golgi network | -2.2265 | -0.328 | HLA-DPB1,HLA-DQB1,HLA-DRB5,LAP3 |
| 5_Member | GO:0031300 | intrinsic component of organelle membrane | -2.21739 | -0.320 | HLA-DPB1,HLA-DQB1,HLA-DRB5,VAMP5,TMEM70 |
| 5_Member | GO:0050867 | positive regulation of cell activation | -2.20452 | -0.312 | MAP3K8,HLA-DPB1,HLA-DQB1,HLA-DRB5,IGKC |
| 5_Member | GO:0045785 | positive regulation of cell adhesion | -2.13366 | -0.253 | MAP3K8,HLA-DPB1,HLA-DQB1,HLA-DRB5,VEGFA |
| 5_Member | GO:0002443 | leukocyte mediated immunity | -2.11346 | -0.237 | BST2,HLA-DPB1,HLA-DQB1,HLA-DRB5,IGKC |
| 5_Member | GO:0022409 | positive regulation of cell-cell adhesion | -2.09382 | -0.225 | MAP3K8,HLA-DPB1,HLA-DQB1,HLA-DRB5 |
| 6_Summary | GO:0008083 | growth factor activity | -5.21996 | -2.359 | AREG,TYMP,OSM,TIMP1,VEGFA,ADA2,STAT1,STAT2,SOCS3,LY6E,TNFSF10,NCF1,ENG,OAS2 |
| 6_Member | GO:0008083 | growth factor activity | -5.21996 | -2.359 | AREG,TYMP,OSM,TIMP1,VEGFA,ADA2 |
| 6_Member | GO:0007259 | receptor signaling pathway via JAK-STAT | -3.96363 | -1.541 | OSM,STAT1,STAT2,VEGFA,SOCS3 |
| 6_Member | GO:0030545 | signaling receptor regulator activity | -3.89049 | -1.498 | AREG,TYMP,LY6E,OSM,TIMP1,VEGFA,TNFSF10,ADA2 |
| 6_Member | GO:0097696 | receptor signaling pathway via STAT | -3.83538 | -1.483 | OSM,STAT1,STAT2,VEGFA,SOCS3 |
| 6_Member | GO:0048018 | receptor ligand activity | -3.39643 | -1.167 | AREG,TYMP,OSM,TIMP1,VEGFA,TNFSF10,ADA2 |
| 6_Member | GO:0030546 | signaling receptor activator activity | -3.35413 | -1.138 | AREG,TYMP,OSM,TIMP1,VEGFA,TNFSF10,ADA2 |
| 6_Member | GO:0005125 | cytokine activity | -3.30197 | -1.093 | AREG,OSM,TIMP1,VEGFA,TNFSF10 |
| 6_Member | GO:0050730 | regulation of peptidyl-tyrosine phosphorylation | -3.07424 | -0.942 | AREG,OSM,VEGFA,SOCS3,NCF1 |
| 6_Member | GO:0005126 | cytokine receptor binding | -3.02349 | -0.902 | ENG,OSM,STAT1,VEGFA,TNFSF10 |
| 6_Member | GO:0061138 | morphogenesis of a branching epithelium | -2.77145 | -0.731 | AREG,ENG,VEGFA,SOCS3 |
| 6_Member | GO:0042509 | regulation of tyrosine phosphorylation of STAT protein | -2.75117 | -0.719 | OSM,VEGFA,SOCS3 |
| 6_Member | GO:0007260 | tyrosine phosphorylation of STAT protein | -2.70856 | -0.689 | OSM,VEGFA,SOCS3 |
| 6_Member | GO:0050731 | positive regulation of peptidyl-tyrosine phosphorylation | -2.68731 | -0.676 | AREG,OSM,VEGFA,NCF1 |
| 6_Member | GO:0001763 | morphogenesis of a branching structure | -2.65501 | -0.647 | AREG,ENG,VEGFA,SOCS3 |
| 6_Member | GO:0018108 | peptidyl-tyrosine phosphorylation | -2.40535 | -0.460 | AREG,OSM,VEGFA,SOCS3,NCF1 |
| 6_Member | GO:0018212 | peptidyl-tyrosine modification | -2.39077 | -0.452 | AREG,OSM,VEGFA,SOCS3,NCF1 |
| 6_Member | GO:0030879 | mammary gland development | -2.15668 | -0.272 | AREG,OAS2,VEGFA |
| 6_Member | GO:0048729 | tissue morphogenesis | -2.1322 | -0.253 | AREG,ENG,LY6E,STAT1,VEGFA,SOCS3 |
| 6_Member | GO:0048754 | branching morphogenesis of an epithelial tube | -2.05905 | -0.200 | AREG,ENG,VEGFA |
| 7_Summary | GO:0042803 | protein homodimerization activity | -4.74201 | -2.043 | BST2,TYMP,ENG,GBP1,HSPB1,STAT1,VEGFA,TRIM22,ADA2,GIMAP7,SPARC,SOCS3,KLF2,TIPARP,RNF213,TIMP1,ZEB2,TNFSF10,IGKC,OAS1,FOS,IFI27 |
| 7_Member | GO:0042803 | protein homodimerization activity | -4.74201 | -2.043 | BST2,TYMP,ENG,GBP1,HSPB1,STAT1,VEGFA,TRIM22,ADA2,GIMAP7 |
| 7_Member | GO:0001568 | blood vessel development | -4.28073 | -1.761 | TYMP,ENG,HSPB1,SPARC,STAT1,VEGFA,SOCS3,KLF2,TIPARP,RNF213 |
| 7_Member | GO:0048514 | blood vessel morphogenesis | -3.89898 | -1.501 | TYMP,ENG,HSPB1,SPARC,STAT1,VEGFA,KLF2,TIPARP,RNF213 |
| 7_Member | GO:0001525 | angiogenesis | -3.59083 | -1.307 | TYMP,ENG,HSPB1,SPARC,STAT1,VEGFA,KLF2,RNF213 |
| 7_Member | GO:0001667 | ameboidal-type cell migration | -3.43956 | -1.198 | ENG,HSPB1,SPARC,STAT1,TIMP1,VEGFA,ZEB2 |
| 7_Member | GO:0045765 | regulation of angiogenesis | -3.39542 | -1.167 | ENG,HSPB1,SPARC,STAT1,VEGFA,KLF2 |
| 7_Member | GO:1901342 | regulation of vasculature development | -3.35598 | -1.138 | ENG,HSPB1,SPARC,STAT1,VEGFA,KLF2 |
| 7_Member | GO:0043122 | regulation of I-kappaB kinase/NF-kappaB signaling | -3.1883 | -1.012 | BST2,HSPB1,STAT1,TNFSF10,TRIM22 |
| 7_Member | GO:0007249 | I-kappaB kinase/NF-kappaB signaling | -2.95353 | -0.860 | BST2,HSPB1,STAT1,TNFSF10,TRIM22 |
| 7_Member | GO:0010631 | epithelial cell migration | -2.49062 | -0.527 | HSPB1,SPARC,STAT1,VEGFA,ZEB2 |
| 7_Member | GO:0090132 | epithelium migration | -2.47522 | -0.520 | HSPB1,SPARC,STAT1,VEGFA,ZEB2 |
| 7_Member | GO:0090130 | tissue migration | -2.4499 | -0.499 | HSPB1,SPARC,STAT1,VEGFA,ZEB2 |
| 7_Member | GO:0010595 | positive regulation of endothelial cell migration | -2.20904 | -0.315 | HSPB1,SPARC,VEGFA |
| 7_Member | GO:0001894 | tissue homeostasis | -2.18074 | -0.291 | HSPB1,IGKC,OAS1,VEGFA |
| 7_Member | GO:0043542 | endothelial cell migration | -2.10968 | -0.236 | HSPB1,SPARC,STAT1,VEGFA |
| 7_Member | GO:0061629 | RNA polymerase II-specific DNA-binding transcription factor binding | -2.0174 | -0.164 | FOS,HSPB1,IFI27,STAT1 |
| 8_Summary | GO:0030218 | erythrocyte differentiation | -4.65468 | -1.994 | HSPA1A,STAT1,VEGFA,ISG15,KLF2,FOS,IFI16,ZFP36L2,GBP1,FGL2 |
| 8_Member | GO:0030218 | erythrocyte differentiation | -4.65468 | -1.994 | HSPA1A,STAT1,VEGFA,ISG15,KLF2 |
| 8_Member | GO:0034101 | erythrocyte homeostasis | -4.50476 | -1.897 | HSPA1A,STAT1,VEGFA,ISG15,KLF2 |
| 8_Member | GO:0002262 | myeloid cell homeostasis | -4.10133 | -1.647 | HSPA1A,STAT1,VEGFA,ISG15,KLF2 |
| 8_Member | GO:0030099 | myeloid cell differentiation | -4.02709 | -1.594 | FOS,HSPA1A,IFI16,STAT1,VEGFA,ISG15,KLF2 |
| 8_Member | GO:0045648 | positive regulation of erythrocyte differentiation | -3.93934 | -1.527 | HSPA1A,STAT1,ISG15 |
| 8_Member | GO:0045639 | positive regulation of myeloid cell differentiation | -3.69254 | -1.374 | FOS,HSPA1A,STAT1,ISG15 |
| 8_Member | GO:0043618 | regulation of transcription from RNA polymerase II promoter in response to stress | -3.51766 | -1.248 | HSPA1A,VEGFA,KLF2 |
| 8_Member | GO:0045646 | regulation of erythrocyte differentiation | -3.4905 | -1.232 | HSPA1A,STAT1,ISG15 |
| 8_Member | GO:0043620 | regulation of DNA-templated transcription in response to stress | -3.36306 | -1.138 | HSPA1A,VEGFA,KLF2 |
| 8_Member | GO:0071356 | cellular response to tumor necrosis factor | -3.35303 | -1.138 | ZFP36L2,GBP1,HSPA1A,STAT1,KLF2 |
| 8_Member | GO:1903706 | regulation of hemopoiesis | -3.24215 | -1.052 | ZFP36L2,FOS,HSPA1A,STAT1,ISG15,FGL2 |
| 8_Member | GO:0034612 | response to tumor necrosis factor | -3.15714 | -0.996 | ZFP36L2,GBP1,HSPA1A,STAT1,KLF2 |
| 8_Member | GO:0048872 | homeostasis of number of cells | -3.00927 | -0.895 | HSPA1A,STAT1,VEGFA,ISG15,KLF2 |
| 8_Member | GO:0045637 | regulation of myeloid cell differentiation | -2.54749 | -0.559 | FOS,HSPA1A,STAT1,ISG15 |
| 9_Summary | GO:0034774 | secretory granule lumen | -4.50067 | -1.897 | MNDA,RNASE2,SPARC,TIMP1,VEGFA,PLAC8,ADA2,BST2 |
| 9_Member | GO:0034774 | secretory granule lumen | -4.50067 | -1.897 | MNDA,RNASE2,SPARC,TIMP1,VEGFA,PLAC8,ADA2 |
| 9_Member | GO:0060205 | cytoplasmic vesicle lumen | -4.47509 | -1.887 | MNDA,RNASE2,SPARC,TIMP1,VEGFA,PLAC8,ADA2 |
| 9_Member | GO:0031983 | vesicle lumen | -4.45818 | -1.877 | MNDA,RNASE2,SPARC,TIMP1,VEGFA,PLAC8,ADA2 |
| 9_Member | GO:0005766 | primary lysosome | -4.11436 | -1.647 | BST2,MNDA,RNASE2,PLAC8,ADA2 |
| 9_Member | GO:0042582 | azurophil granule | -4.11436 | -1.647 | BST2,MNDA,RNASE2,PLAC8,ADA2 |
| 9_Member | GO:0035578 | azurophil granule lumen | -3.91635 | -1.514 | MNDA,RNASE2,PLAC8,ADA2 |
| 9_Member | GO:0005775 | vacuolar lumen | -2.85166 | -0.783 | MNDA,RNASE2,PLAC8,ADA2 |
| 10_Summary | GO:0046686 | response to cadmium ion | -4.41154 | -1.838 | FOS,MT2A,SPARC,NCF1,AREG,HSPA1A,HSPB1,STAT1,KLF2 |
| 10_Member | GO:0046686 | response to cadmium ion | -4.41154 | -1.838 | FOS,MT2A,SPARC,NCF1 |
| 10_Member | GO:0071276 | cellular response to cadmium ion | -3.72677 | -1.402 | FOS,MT2A,NCF1 |
| 10_Member | GO:0006979 | response to oxidative stress | -3.5975 | -1.310 | AREG,FOS,HSPA1A,HSPB1,STAT1,KLF2,NCF1 |
| 10_Member | GO:0034599 | cellular response to oxidative stress | -2.89296 | -0.811 | FOS,HSPA1A,HSPB1,KLF2,NCF1 |
| 10_Member | GO:0062197 | cellular response to chemical stress | -2.59727 | -0.596 | FOS,HSPA1A,HSPB1,KLF2,NCF1 |
| 10_Member | GO:0034614 | cellular response to reactive oxygen species | -2.03598 | -0.178 | FOS,KLF2,NCF1 |
| 11_Summary | GO:0043086 | negative regulation of catalytic activity | -4.19725 | -1.690 | BST2,IFI6,HSPB1,IFI16,RGS2,TIMP1,VEGFA,SOCS3,CDC42SE1,MIDN,PSMB9,TNFSF10,HSPA1A,RNF144B,SPARC,FOS |
| 11_Member | GO:0043086 | negative regulation of catalytic activity | -4.19725 | -1.690 | BST2,IFI6,HSPB1,IFI16,RGS2,TIMP1,VEGFA,SOCS3,CDC42SE1,MIDN |
| 11_Member | GO:0052548 | regulation of endopeptidase activity | -3.68692 | -1.374 | BST2,IFI6,IFI16,PSMB9,TIMP1,VEGFA,TNFSF10 |
| 11_Member | GO:0030162 | regulation of proteolysis | -3.63467 | -1.340 | BST2,IFI6,HSPA1A,IFI16,PSMB9,TIMP1,VEGFA,TNFSF10,RNF144B |
| 11_Member | GO:0052547 | regulation of peptidase activity | -3.51713 | -1.248 | BST2,IFI6,IFI16,PSMB9,TIMP1,VEGFA,TNFSF10 |
| 11_Member | GO:2000116 | regulation of cysteine-type endopeptidase activity | -3.27698 | -1.080 | IFI6,IFI16,PSMB9,VEGFA,TNFSF10 |
| 11_Member | GO:0051346 | negative regulation of hydrolase activity | -3.18762 | -1.012 | BST2,IFI6,IFI16,RGS2,TIMP1,VEGFA |
| 11_Member | GO:0010951 | negative regulation of endopeptidase activity | -3.16488 | -0.998 | BST2,IFI6,IFI16,TIMP1,VEGFA |
| 11_Member | GO:0010466 | negative regulation of peptidase activity | -3.08902 | -0.949 | BST2,IFI6,IFI16,TIMP1,VEGFA |
| 11_Member | GO:0031093 | platelet alpha granule lumen | -3.06447 | -0.938 | SPARC,TIMP1,VEGFA |
| 11_Member | GO:2000117 | negative regulation of cysteine-type endopeptidase activity | -2.75117 | -0.719 | IFI6,IFI16,VEGFA |
| 11_Member | GO:0007565 | female pregnancy | -2.68731 | -0.676 | FOS,RGS2,TIMP1,VEGFA |
| 11_Member | GO:0031091 | platelet alpha granule | -2.681 | -0.672 | SPARC,TIMP1,VEGFA |
| 11_Member | GO:0045861 | negative regulation of proteolysis | -2.53241 | -0.555 | BST2,IFI6,IFI16,TIMP1,VEGFA |
| 11_Member | GO:0044706 | multi-multicellular organism process | -2.48253 | -0.522 | FOS,RGS2,TIMP1,VEGFA |
| 11_Member | GO:0004857 | enzyme inhibitor activity | -2.32919 | -0.404 | BST2,HSPB1,TIMP1,SOCS3,CDC42SE1 |
| 12_Summary | GO:1901652 | response to peptide | -4.08441 | -1.640 | AREG,SPARC,STAT1,STAT2,TIMP1,TNFSF10,SOCS3,KLF2,ZFP36L2,ENG,FOS,DDX17,NCF1,MT2A,RGS2 |
| 12_Member | GO:1901652 | response to peptide | -4.08441 | -1.640 | AREG,SPARC,STAT1,STAT2,TIMP1,TNFSF10,SOCS3,KLF2 |
| 12_Member | GO:0031960 | response to corticosteroid | -3.98793 | -1.560 | AREG,ZFP36L2,ENG,FOS,SPARC |
| 12_Member | GO:0051591 | response to cAMP | -3.8618 | -1.497 | AREG,FOS,SPARC,STAT1 |
| 12_Member | GO:0043434 | response to peptide hormone | -3.80549 | -1.462 | AREG,SPARC,STAT1,STAT2,TIMP1,TNFSF10,SOCS3 |
| 12_Member | GO:0048545 | response to steroid hormone | -3.44244 | -1.198 | AREG,ZFP36L2,ENG,FOS,SPARC,DDX17 |
| 12_Member | GO:0000302 | response to reactive oxygen species | -3.40561 | -1.171 | AREG,FOS,STAT1,KLF2,NCF1 |
| 12_Member | GO:0046683 | response to organophosphorus | -3.29837 | -1.093 | AREG,FOS,SPARC,STAT1 |
| 12_Member | GO:0051384 | response to glucocorticoid | -3.11195 | -0.961 | AREG,ZFP36L2,FOS,SPARC |
| 12_Member | GO:0014074 | response to purine-containing compound | -3.10104 | -0.956 | AREG,FOS,SPARC,STAT1 |
| 12_Member | GO:0010035 | response to inorganic substance | -2.97165 | -0.865 | AREG,FOS,MT2A,SPARC,STAT1,KLF2,NCF1 |
| 12_Member | GO:0097305 | response to alcohol | -2.27392 | -0.363 | FOS,RGS2,SPARC,KLF2 |
| 12_Member | GO:0042542 | response to hydrogen peroxide | -2.10675 | -0.236 | AREG,STAT1,KLF2 |
| 12_Member | GO:0016525 | negative regulation of angiogenesis | -2.08263 | -0.216 | SPARC,STAT1,KLF2 |
| 12_Member | GO:2000181 | negative regulation of blood vessel morphogenesis | -2.06685 | -0.205 | SPARC,STAT1,KLF2 |
| 12_Member | GO:1901343 | negative regulation of vasculature development | -2.05905 | -0.200 | SPARC,STAT1,KLF2 |
| 13_Summary | GO:0040008 | regulation of growth | -4.05405 | -1.616 | BST2,DUSP6,HSPA1A,MT2A,OSM,RGS2,VEGFA,SOCS3,PLAC8,AREG,LY6E,KLF2 |
| 13_Member | GO:0040008 | regulation of growth | -4.05405 | -1.616 | BST2,DUSP6,HSPA1A,MT2A,OSM,RGS2,VEGFA,SOCS3,PLAC8 |
| 13_Member | GO:0045926 | negative regulation of growth | -3.1883 | -1.012 | BST2,HSPA1A,MT2A,RGS2,PLAC8 |
| 13_Member | GO:0048589 | developmental growth | -2.75238 | -0.719 | AREG,DUSP6,LY6E,RGS2,VEGFA,KLF2,PLAC8 |
| 14_Summary | GO:0022626 | cytosolic ribosome | -3.72495 | -1.402 | RPLP0,RPS4Y1,RPS26,ISG15,HSPA1A,RBM3,DDX17,NOP10,ZFP36L2,HSPB1,RGS2 |
| 14_Member | GO:0022626 | cytosolic ribosome | -3.72495 | -1.402 | RPLP0,RPS4Y1,RPS26,ISG15 |
| 14_Member | GO:0022627 | cytosolic small ribosomal subunit | -3.66331 | -1.358 | RPS4Y1,RPS26,ISG15 |
| 14_Member | GO:0043484 | regulation of RNA splicing | -3.11195 | -0.961 | HSPA1A,RBM3,RPS26,DDX17 |
| 14_Member | GO:1990904 | ribonucleoprotein complex | -3.01548 | -0.897 | HSPA1A,RBM3,RPLP0,RPS4Y1,RPS26,ISG15,DDX17,NOP10 |
| 14_Member | GO:0015935 | small ribosomal subunit | -2.99124 | -0.882 | RPS4Y1,RPS26,ISG15 |
| 14_Member | GO:0044391 | ribosomal subunit | -2.79769 | -0.745 | RPLP0,RPS4Y1,RPS26,ISG15 |
| 14_Member | GO:0003735 | structural constituent of ribosome | -2.78889 | -0.738 | RPLP0,RPS4Y1,RPS26,ISG15 |
| 14_Member | GO:0006412 | translation | -2.36679 | -0.433 | ZFP36L2,HSPB1,RBM3,RGS2,RPLP0,RPS4Y1,RPS26 |
| 14_Member | GO:0005840 | ribosome | -2.3484 | -0.420 | RPLP0,RPS4Y1,RPS26,ISG15 |
| 14_Member | GO:0043043 | peptide biosynthetic process | -2.28862 | -0.373 | ZFP36L2,HSPB1,RBM3,RGS2,RPLP0,RPS4Y1,RPS26 |
| 15_Summary | GO:0031400 | negative regulation of protein modification process | -3.23198 | -1.044 | DUSP6,ENG,HSPA1A,HSPB1,RGS2,SOCS3,ISG15,GBP1,STAT1,RIPOR2,MIDN |
| 15_Member | GO:0031400 | negative regulation of protein modification process | -3.23198 | -1.044 | DUSP6,ENG,HSPA1A,HSPB1,RGS2,SOCS3,ISG15 |
| 15_Member | GO:1902532 | negative regulation of intracellular signal transduction | -3.16861 | -0.998 | DUSP6,GBP1,HSPA1A,HSPB1,RGS2,STAT1,RIPOR2 |
| 15_Member | GO:0042326 | negative regulation of phosphorylation | -3.15209 | -0.993 | DUSP6,ENG,HSPB1,RGS2,SOCS3,MIDN |
| 15_Member | GO:0045936 | negative regulation of phosphate metabolic process | -2.84899 | -0.783 | DUSP6,ENG,HSPB1,RGS2,SOCS3,MIDN |
| 15_Member | GO:0010563 | negative regulation of phosphorus metabolic process | -2.844 | -0.780 | DUSP6,ENG,HSPB1,RGS2,SOCS3,MIDN |
| 15_Member | GO:0001933 | negative regulation of protein phosphorylation | -2.56992 | -0.576 | DUSP6,ENG,HSPB1,RGS2,SOCS3 |
| 16_Summary | GO:0071560 | cellular response to transforming growth factor beta stimulus | -3.14944 | -0.993 | ZFP36L2,ENG,FOS,HSPA1A,ZEB2,DUSP6,HSPB1,VEGFA,RPLP0,LAP3,IGKC |
| 16_Member | GO:0071560 | cellular response to transforming growth factor beta stimulus | -3.14944 | -0.993 | ZFP36L2,ENG,FOS,HSPA1A,ZEB2 |
| 16_Member | GO:0071559 | response to transforming growth factor beta | -3.10392 | -0.956 | ZFP36L2,ENG,FOS,HSPA1A,ZEB2 |
| 16_Member | GO:0070848 | response to growth factor | -3.06926 | -0.940 | ZFP36L2,DUSP6,ENG,FOS,HSPA1A,HSPB1,VEGFA,ZEB2 |
| 16_Member | GO:0007179 | transforming growth factor beta receptor signaling pathway | -2.61565 | -0.612 | ENG,FOS,HSPA1A,ZEB2 |
| 16_Member | GO:0071363 | cellular response to growth factor stimulus | -2.52349 | -0.550 | ZFP36L2,ENG,FOS,HSPA1A,HSPB1,VEGFA,ZEB2 |
| 16_Member | GO:0017015 | regulation of transforming growth factor beta receptor signaling pathway | -2.25469 | -0.350 | ENG,HSPA1A,ZEB2 |
| 16_Member | GO:1903844 | regulation of cellular response to transforming growth factor beta stimulus | -2.22707 | -0.328 | ENG,HSPA1A,ZEB2 |
| 16_Member | GO:0005925 | focal adhesion | -2.20026 | -0.309 | ENG,HSPA1A,HSPB1,RPLP0,LAP3 |
| 16_Member | GO:0030055 | cell-substrate junction | -2.17075 | -0.284 | ENG,HSPA1A,HSPB1,RPLP0,LAP3 |
| 16_Member | GO:0072562 | blood microparticle | -2.10675 | -0.236 | ENG,HSPA1A,IGKC |
| 17_Summary | GO:0034655 | nucleobase-containing compound catabolic process | -2.99989 | -0.889 | ZFP36L2,TYMP,HSPA1A,OAS2,RNASE2,ADA2 |
| 17_Member | GO:0034655 | nucleobase-containing compound catabolic process | -2.99989 | -0.889 | ZFP36L2,TYMP,HSPA1A,OAS2,RNASE2,ADA2 |
| 17_Member | GO:0046700 | heterocycle catabolic process | -2.77551 | -0.731 | ZFP36L2,TYMP,HSPA1A,OAS2,RNASE2,ADA2 |
| 17_Member | GO:0044270 | cellular nitrogen compound catabolic process | -2.74693 | -0.717 | ZFP36L2,TYMP,HSPA1A,OAS2,RNASE2,ADA2 |
| 17_Member | GO:0019439 | aromatic compound catabolic process | -2.69566 | -0.678 | ZFP36L2,TYMP,HSPA1A,OAS2,RNASE2,ADA2 |
| 17_Member | GO:1901361 | organic cyclic compound catabolic process | -2.55015 | -0.560 | ZFP36L2,TYMP,HSPA1A,OAS2,RNASE2,ADA2 |
| 17_Member | GO:0006401 | RNA catabolic process | -2.10968 | -0.236 | ZFP36L2,HSPA1A,OAS2,RNASE2 |
| 18_Summary | GO:0042058 | regulation of epidermal growth factor receptor signaling pathway | -2.95626 | -0.860 | AREG,IFI6,NCF1,HSPB1,VEGFA,SOCS3,TIPARP,RGS2,MIDN |
| 18_Member | GO:0042058 | regulation of epidermal growth factor receptor signaling pathway | -2.95626 | -0.860 | AREG,IFI6,NCF1 |
| 18_Member | GO:1901184 | regulation of ERBB signaling pathway | -2.85713 | -0.784 | AREG,IFI6,NCF1 |
| 18_Member | GO:0007169 | transmembrane receptor protein tyrosine kinase signaling pathway | -2.74455 | -0.717 | AREG,IFI6,HSPB1,VEGFA,SOCS3,TIPARP,NCF1 |
| 18_Member | GO:0007173 | epidermal growth factor receptor signaling pathway | -2.45891 | -0.505 | AREG,IFI6,NCF1 |
| 18_Member | GO:0038127 | ERBB signaling pathway | -2.32197 | -0.398 | AREG,IFI6,NCF1 |
| 18_Member | GO:0043549 | regulation of kinase activity | -2.27947 | -0.365 | AREG,HSPB1,RGS2,VEGFA,SOCS3,MIDN,NCF1 |
| 19_Summary | GO:0005770 | late endosome | -2.94669 | -0.857 | BST2,HLA-DRB5,SIGLEC1,IFITM3,VAMP5 |
| 19_Member | GO:0005770 | late endosome | -2.94669 | -0.857 | BST2,HLA-DRB5,SIGLEC1,IFITM3,VAMP5 |
| 20_Summary | GO:0000165 | MAPK cascade | -2.86511 | -0.790 | ZFP36L2,MAP3K8,DUSP6,GBP1,OSM,RGS2,VEGFA,NCF1,AREG,STAT1,FGL2,FOS,IFI16,NFIL3 |
| 20_Member | GO:0000165 | MAPK cascade | -2.86511 | -0.790 | ZFP36L2,MAP3K8,DUSP6,GBP1,OSM,RGS2,VEGFA,NCF1 |
| 20_Member | GO:0045596 | negative regulation of cell differentiation | -2.54449 | -0.558 | AREG,ZFP36L2,GBP1,RGS2,STAT1,VEGFA,FGL2 |
| 20_Member | GO:0002521 | leukocyte differentiation | -2.40412 | -0.460 | ZFP36L2,FOS,IFI16,NFIL3,VEGFA,FGL2 |
| 20_Member | GO:1903131 | mononuclear cell differentiation | -2.17912 | -0.291 | ZFP36L2,IFI16,NFIL3,VEGFA,FGL2 |
